# The Efficacy and Safety of Simple-Needling Therapy for Treating Ankylosing Spondylitis: A Systematic Review and Meta-Analysis of Randomized Controlled Trials

**DOI:** 10.1155/2020/4276380

**Published:** 2020-06-13

**Authors:** Yichen Xuan, Hui Huang, Yiyong Huang, Duanyong Liu, Xiuwu Hu, Lele Geng

**Affiliations:** ^1^Science and Technology College of Jiangxi University of Traditional Chinese Medicine, Nanchang, China; ^2^Department of Acupuncture, Nanchang Hongdu Hospital of Traditional Chinese Medicine, Nanchang, China

## Abstract

**Background:**

Clinical investigators have found that the use of needling in the treatment of ankylosing spondylitis (AS) has a good clinical application prospect in recent years. However, these studies were insufficient to provide evidence for the efficacy and safety of simple-needling for AS. So, we performed a systematic review and meta-analysis to evaluate the efficacy and safety of simple-needling for treating AS.

**Methods:**

We searched the PubMed, Cochrane Library, Embase, China National Knowledge Infrastructure (CNKI), Chinese Biomedical Literature Database (CBM), Wangfang database (Wanfang), Chinese Science and Technology Periodical Database (VIP), and any other gray literature sources for randomized controlled trials (RCTs) that used simple-needling to treat AS before June 2019 with the language restriction of Chinese and English. Researchers evaluated the retrieved literature studies and extracted valid data according to relevant requirements and used RevMan5.3 software for meta-analysis.

**Results:**

A total of 10 studies were included, all of which were Chinese literature studies, involving 729 patients. Compared with the control groups, simple-needling groups had a better effect on the clinical effective rate (RR = 1.20, 95% CI (1.11, 1.29), *P* < 0.00001), TCM syndrome score (MD = −5.26, 95% CI (−5.99, −4.53), *P* < 0.00001), symptom score (MD = −8.08, 95% CI (−10.18, −5.97), *P* < 0.00001), and Schober test outcome (MD = 0.39, 95% CI (0.15, 0.64), *P*=0.002). Sensibility analysis was based on the leave-one-out cross-validation procedure, and the results showed no significant changes. Most studies did not describe adverse reactions. The funnel plot suggested publication bias on clinical effectiveness.

**Conclusions:**

This systematic review and meta-analysis demonstrated that simple-needling was effective as an intervention for AS. However, due to the low quality of the methodology of included studies, the designs of clinical trials were not rigorously standardized. Therefore, it is necessary to carry out multiquality RCTs for verification.

## 1. Introduction

Ankylosing spondylitis (AS) is the main form of chronic inflammatory arthritis affecting the axial skeleton and is characterized by excess spinal bone formation, inflammatory back pain, radiographic sacroiliitis, and a high prevalence of HLA-B27 [1, 2]. In 2014, the estimated number of patients with AS was from 1.30 to 1.56 million in Europe and from 4.63 to 4.98 million in Asia [3]. In 2017, a study enrolled 1251 AS patients from China. The results showed that the mean age of onset and diagnosis was 29.2 and 33.5 years, and the ratio of male to female was 2.7 : 1 [4]. A recent study reported that the risk of cardiovascular events in AS patients increased by 30–50% compared with the general population [5]. In addition, patients with AS had increased risks of multiple diseases such as chronic obstructive pulmonary disease, asthma, type 2 diabetes, stroke, cancer, and depression [6–13]. The occurrence of these comorbidities made us have to pay attention to the treatment of AS. The American College of Rheumatology and Spondylitis Association of America recommended that nonsteroidal anti-inflammatory drugs (NSAIDs) and tumor necrosis factor inhibitors (TNFi) remain the primary classes of medications for the treatment of AS in 2019 [14]. Common adverse reactions to medications such as abdominal pain, loss of appetite, upper gastrointestinal bleeding, and other important factors restrict the treatment of some patients. Furthermore, the cost of medicine must be considered for patients. For example, TNFi therapy can effectively improve joint mobility in patients with AS [15] and delay patients' spine imaging changes [16]. However, the cost of medicine has brought a large financial burden to AS patients and their families [17]. Needling, as a traditional Chinese therapy, is a popular complementary and alternative therapy in the world [18, 19]. Due to limited evidence to support the effectiveness of needling for AS, needling is not a standard treatment for AS. But as an unconventional therapy, needling is the choice of many patients [20]. In order to obtain more effective treatment for patients, needling combined with other therapies had been commonly used in China to treat AS, such as needling combined with moxibustion, traditional Chinese medicine (TCM), and sulfasalazine [21–23]. The efficacy and superiority of needling combined with other therapies for AS patients had been evaluated and verified in the meta-analysis [24–27]. Considering that the therapeutic effects of simple-needling therapy could not be distinguished. Therefore, to evaluate the efficacy and safety of simple-needling therapy, we comprehensively collected RCTs of simple-needling therapy in the treatment of AS and conducted a systematic review and meta-analysis.

## 2. Data and Method

### 2.1. Eligibility Criteria

#### 2.1.1. Types of Studies

The included studies were RCTs in Chinese and English.

#### 2.1.2. Types of Participants

All participants were clearly diagnosed as AS, and there were no restrictions on the gender, age, and duration of the study subjects.

#### 2.1.3. Types of Interventions

Interventions in the experimental groups included the use of needling therapy alone. Trials that used a combination of other interventions were excluded, such as other Chinese medicines, Western medicine, moxibustion, and Tuina. The interventions in the control groups were nonneedling treatments.

#### 2.1.4. Types of Outcome Measures

Included trials must report outcome indicators in at least one of the following forms: (1) clinical effective rate, the proportion of patients including improvement, effectiveness, and cure, according to the efficacy standards of each clinical study; (2) TCM syndrome score; (3) symptom score; (4) Schober test outcome; and (5) adverse reactions.

### 2.2. Exclusion Criteria

Exclusion criteria were as follows: (1) nonclinical RCTs, such as animal experiments, molecular experiments, clinical experience, and research progress; (2) repeated publication and trials with the same original data; (3) inability to obtain the original text, and data that could not be extracted from the tests; (4) research designs that were unreasonable; and (5) the intervention measures of the experimental groups that were moxibustion or acupotomy.

### 2.3. Search Strategy

We searched the following electronic databases by computer: PubMed, the Cochrane Library, Embase, China National Knowledge Infrastructure (CNKI), Chinese Biomedical Literature Database (CBM), Wanfang database (WF), and Chinese Science and Technology Periodical Database (VIP). We searched from inception to June 2019. The search was carried out by means of a combination of Medical Subject Heading (MeSH) terms and free words. The search terms were ankylosing spondylitis, acupuncture, acupuncture therapy, needle, needling, and randomized. We also retrieved any other gray literature sources.

### 2.4. Screening of Literature Studies

All the searched literature titles were retrieved into EndnoteX9, manual auxiliary operation combined with EndnoteX9 to review the literature, and eliminated the duplicate publication of literature. Two researchers separately read the titles and abstracts and deleted the documents that were obviously unqualified according to the inclusion and exclusion criteria. The rest of the literature were read in full text and finally confirmed the included literature. If there were different opinions between the two researchers in the process of literature screening, they would need to be resolved through negotiation. If the outcome of the consultation was not reached, it would be determined by the third researcher.

### 2.5. Data Extraction

The included literature studies were read independently by two researchers who extracted the data. The extraction included the authors' name, year of publication, literature sources, sample size, baselines, research designs, diagnostic criteria, random methods, distribution concealment, blind implementation, missing or shedding reports, interventions, courses of treatment, efficacy criteria, efficacy indicators, and adverse events. If the information about the original studies was missing or unclear, we would contact the lead author by e-mail or phone as far as possible.

### 2.6. Quality Assessment

Based on the bias risk assessment method in the Cochrane handbook [28], two researchers independently analyzed the quality of the included literature. If there were differences, they would defer to third-party opinions and reach an agreement. The evaluation included (1) random sequence generation, (2) allocation concealment, (3) blinding implementation, (4) incomplete outcome data, (5) selective reporting, and (6) other sources of bias.

### 2.7. Statistical Analysis

RevMan5.3 software was used for data analysis. The results were analyzed to evaluate the difference between needling therapy and control groups. The mean difference (MD) was used to represent the measurement data, and the relative risk (RR) was used to represent the counting data. Each effect was expressed by a 95% confidence interval (CI), and the difference was statistically significant (*P* < 0.05). Heterogeneity within RCTs was examined based on the *I*^2^ test. *I*^2^ ≤ 50% was considered that there was low heterogeneity between the studies. Meta-analysis was carried out by using the fixed effect model. 50% < *I*^2^ < 75% was considered that there was moderate heterogeneity, and *I*^2^ ≥ 75% was considered that there was significant heterogeneity. Meta-analysis was carried out by using a random effect model, and its heterogeneity sources were analyzed. If the data could not be merged, the descriptive analysis would be used.

### 2.8. Sensitivity Analysis

Sensitivity analysis was based on the leave-one-out cross-validation procedure to clarify the stability and reliability of the results.

### 2.9. Publication of Bias Risk Assessment

If the number of studies for pooling was not less than 8, publication bias was assessed using a funnel plot.

## 3. Result

### 3.1. Screened Results of Literature Studies

**Figure 1:**
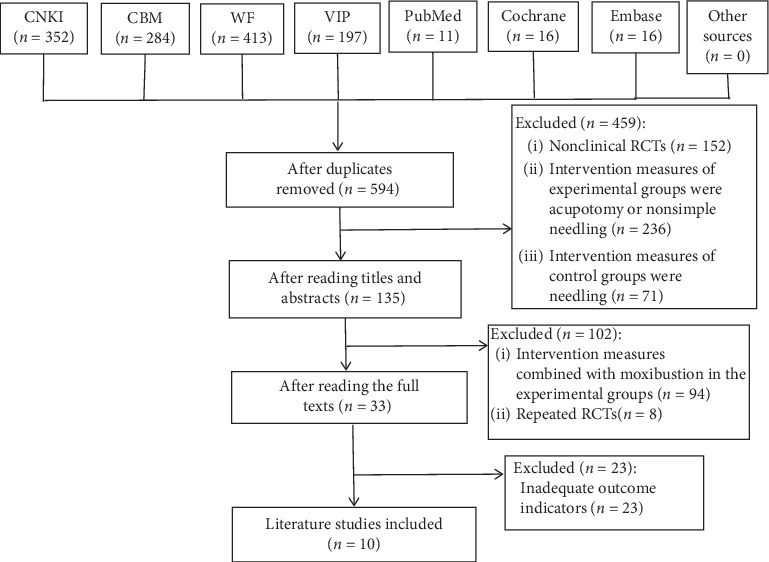
Flow diagram of screening literature studies.

A total of 1289 articles, 43 English literature studies and 1246 Chinese literature studies, were searched. After screening, a total of 10 studies [29–38] were included, all of which were Chinese literature studies (Figure 1).

### 3.2. Characteristics of Included Literature Studies

A total of 729 patients, 377 patients in the experimental groups and 352 patients in the control groups, were enrolled in 10 RCTs. The intervention measures of the experimental groups were simple-needling, while those of the control groups were the routine treatment of Western medicine (Table 1).

### 3.3. Diagnostic Criteria of Included Studies

Two studies [30, 37] referred to Western medicine diagnostic criteria and TCM diagnostic criteria, respectively. The diagnostic criteria for Western medicine were the New York standards revised in 1984, and the diagnostic criteria for TCM were the *Principles for Clinical Research of New* TCM in 2002 and Wang Yongyan's *Internal Medicine for TCM* in 2001. The remaining eight studies [29, 31–36, 38] referred to Western medicine diagnostic criteria: four studies [31, 32, 34, 35] were based on the New York Standard revised in 1984, one study [29] was based on the Guideline for the Diagnosis and Treatment of AS with Chinese Medical Association Rheumatology Branch in 2010, one study [36] was based on the diagnostic criteria developed by the Academic Conference on Integrative Medicine for Rheumatology in 1984, one study [38] was based on the New York Standard integrated with the Academic Conference on Integrative Medicine for Rheumatology in 1984, and one study [33] did not describe the diagnostic criteria.

### 3.4. Efficacy Criteria of Included Studies

Eight studies [29, 30, 32–34, 36–38] described the efficacy criteria of the RCTs: two studies [33, 34] referred to the *Diagnostic and Efficacy Standards for TCM Diseases* promulgated by the Chinese Medicine Administration in 1994, one study [29] referred to the *Principles for Clinical Research of New* TCM in 2002, one study [32] was based on the National Rheumatology Conference in 1998, one study [37] referred to Wang Yongyan's *Internal Medicine for TCM* in 2001, and three studies [30, 36, 38] were based on self-made criteria. The remaining 2 studies [31, 35]did not address the efficacy criteria.

### 3.5. Quality Evaluation of Included Studies

**Figure 2:**
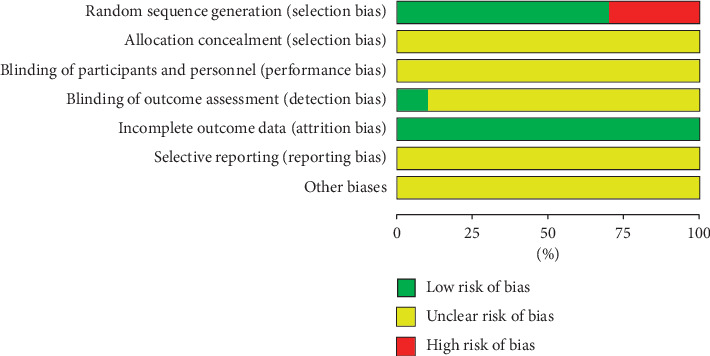
Risk of bias graph.

**Figure 3:**
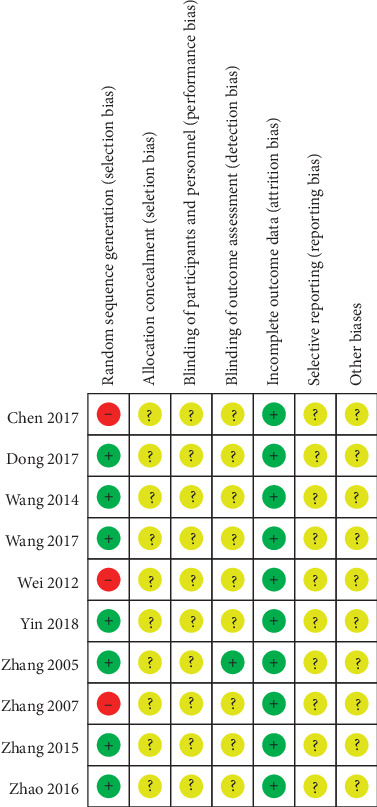
Risk of bias summary.

The quality of the 10 studies was low. 7 studies [29, 31–35, 38] reported the random sequence generation, and 3 studies [30, 36, 37] had only random words. None of the 10 studies [29–38] described the implementation of allocation concealment. One study [38] described the implementation of single blindness, and the remaining 9 studies [29–37] did not describe the implementation of blinding. The final outcomes reported in the 10 studies [29–38] were consistent with the outcomes they set, so the outcome data were complete. The information for all studies was insufficient to determine whether selective reports exist and failed to obtain other sources of bias (Figures 2 and 3).

### 3.6. Meta-Analysis

#### 3.6.1. Clinical Effective Rate

**Figure 4:**
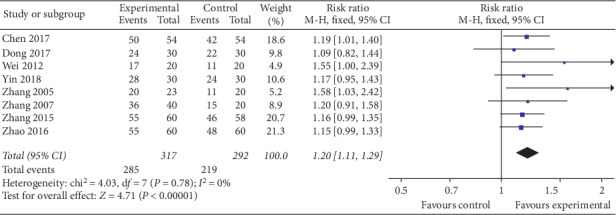
Forest plot of experimental groups versus control groups: clinical effective rate.

The combined results of 8 trials [29, 30, 32–34, 36–38] released the idea that simple-needling group was better than the control group statistically in the clinical effective rate (RR = 1.20, 95% CI (1.11, 1.29), *P* < 0.00001). Low heterogeneity between the studies was found (*I*^2^ = 0%) using the fixed-effect model (Figure 4).

#### 3.6.2. TCM Syndrome Score

**Figure 5:**
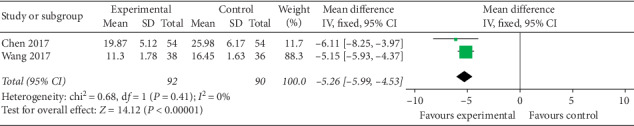
Forest plot of experimental groups versus control groups: TCM syndrome score.

There was a significant difference in TCM syndrome score according to the combined results of 2 studies [30, 31] (MD = −5.26, 95% CI (−5.99, −4.53), *P* < 0.00001). According to the heterogeneity test, there was low heterogeneity between the 2 studies (*I*^2^ = 0%), using the fixed-effect model.  Figure 5.

#### 3.6.3. Symptom Score

**Figure 6:**
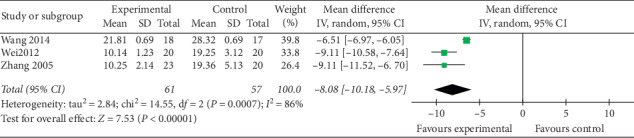
Forest plot of experimental groups versus control groups: symptom score.

There were 3 RCTs [35, 36, 38] for meta-analysis that compared the outcome of symptom score. The result showed a significant difference between the simple-needling group and the control group (MD = −8.08, 95% CI (−10.18, −5.97), *P* < 0.00001). The random-effect model was used owing to their significant heterogeneity (*I*^2^ = 86%). We reviewed the original literature studies to infer that the sources of heterogeneity may be related to different scoring criteria or the difference between patients or different operators (Figure 6).

#### 3.6.4. Schober Test Outcome

**Figure 7:**
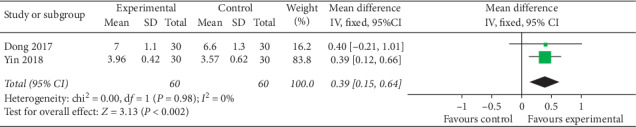
Forest plot of experimental groups versus control groups: Schober test outcome.

Two RCTs [29, 32] reported the Schober test outcome, and a significant difference between the two interventions was found from the combined results indicated (MD = 0.39, 95% CI (0.15, 0.64), *P*=0.002). There was low heterogeneity between the 2 studies (*I*^2^ = 0%), using the fixed-effect model.  Figure 7.

### 3.7. Sensibility Analysis

We conducted the sensibility analysis based on the leave-one-out cross-validation procedure, and the results showed no significant changes, indicating that the meta-analysis results were more reliable.

### 3.8. Safety Evaluation

Only 2 studies [31, 35] described adverse reactions, and there were no obvious adverse reactions in the experimental groups.

### 3.9. Publication Bias

**Figure 8:**
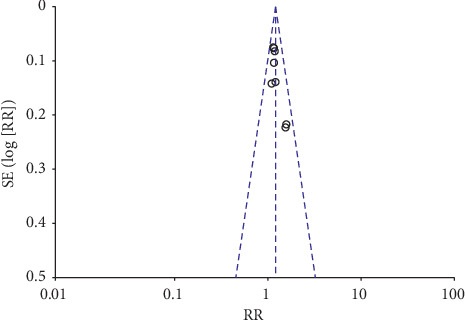
Clinical effective rate funnel plot.

A meta-analysis of the clinical effectiveness of the 8 studies [29, 30, 32–34, 36–38] and a funnel plot showed asymmetrical and skewed distributions. This may have publication bias (Figure 8).

## 4. Discussion

### 4.1. Needling Treatment of the Potential of AS

AS is a chronic inflammatory autoimmune disease that mainly causes lumbosacral pain and stiffness and finally affects the entire spine [1]. At present, adverse reactions and financial burdens are caused to patients by current medication treatment to a certain extent, and needling therapy as a supplementary replacement therapy has attracted the attention of scholars because of its unique curative effects and low side effects [39]. From the perspective of modern medicine, needling therapy has an obvious analgesic effect [40] and improves the immune function of the bodies [41]. From the perspective of traditional meridian theory, it is considered that kidney deficiency is the basis of AS generation, and kidney essence can be supplemented by needling therapy [42]. Studies had shown that needling therapy could effectively alleviate spinal pain, improve spinal function, and significantly reduce the levels of TNF-*α* and IL-1*β* inflammatory factors in patients with AS [43–45].

### 4.2. Systematic Review and Meta-Analysis Main Findings

In this review, we identified 10 studies of simple-needling for AS, and these studies included 729 participants. All the interventions included in the studies were compared with simple-needling treatment and conventional Western medicine treatment. The results showed that the clinical effective rate of simple-needling was higher than that of Western medicine, and it was better than the intervention of control groups in improving TCM syndrome score, symptom score, and spinal function.

### 4.3. Systematic Review and Meta-Analysis Advantages

The meta-analysis was proposed by the American educator Glass in 1976 [46]. Its essence is to qualitatively or quantitatively synthesize multiple independent studies, solve clinical divergence opinions, and enhance the reliability and objectivity of clinical application effects. Meta-analysis has been widely used by various clinical medicine majors in recent years [47]. This is the first meta-analysis and systematic review of simple-needling for the intervention of AS. The use of English and Chinese databases allowed access to publications in common languages for needling research and retrieved any other gray literature sources. Through the meta-analysis, we analyzed the data of the included studies to clarify that the simple-needling is effective as an intervention for AS and to provide a more reliable basis for clinical decision-making.

### 4.4. Systematic Review and Meta-Analysis Limitations

This systematic review and meta-analysis had several limitations. (1) Although we had retrieved a large number of Chinese and English studies, the final included studies were published in Chinese. The patients included were all from China. (2) The sample size included in the studies was small, and most studies did not describe case shedding. Only two studies described case shedding, but there was no intentional analysis. (3) In the included studies, simple-needling therapy was used as an experimental group compared with conventional Western medicine treatment. The results of each study were positive for simple-needling treatment. We may miss some negative data, which could lead to an overestimate of the effectiveness of simple-needling for AS. (4) The diagnostic criteria and efficacy criteria of RCTs were inconsistent and may result in selection and measurement bias. (5) 7 studies described random methods, 3 studies had only random words, but the details of randomization procedures were often absent. None of the RCTs described the implementation of allocation concealment. One study described the implementation of single blindness, and the remaining 9 studies did not describe, which may pose risks of bias. (6) The widely validated and accepted outcome measures were not used in most of the studies we included, which may have some impact on the results of the meta-analysis. (7) All trials did not describe the follow-up; only to determine the short-term efficacy of simple-needling, its long-term efficacy was not judged. (8) Only 2 studies evaluated safety, and the rest of the studies were unable to judge their safety. (9) Although this study used a funnel plot to describe the publication bias of the clinical efficacy of simple-needling in the treatment of AS, the result may be affected by the lack of inclusion of the studies. All of the above restrictions may cause instability of the meta-analysis results.

## 5. Conclusion

Through comprehensive analysis, this study demonstrated that simple-needling was effective as an intervention for AS. However, there were few relevant studies. Including the low quality of the research methodology, the sample size was small, and the diagnostic criteria and the efficacy criteria were inconsistent, so the results were not representative. Therefore, more rigorous RCTs are needed to verify the above conclusions. In the future, such research should be carried out in accordance with the requirements of evidence-based medicine. The top-level design of research programs, random methods, allocation of hidden methods, implementation of blind methods, adverse reactions, and follow-up data should be valued. It is best to use the latest internationally recognized diagnostic criteria and efficacy criteria. Meanwhile, we used the outcome indicators that are widely validated and accepted at the international level and tried to compare them with other interventions, to further clarify the therapeutic advantages of simple-needling for AS and provide a reliable and objective reference for the clinical treatment of AS.

## Figures and Tables

**Table 1:** Characteristics of included literature studies.

Study ID	Location	Sample size		Intervention		Duration of treatment	Shedding case		Lost case	Outcome indicator
		Experimental	Control	Experimental	Control		Experimental	Control		
Yin 2018 [29]	China	30	30	Internal heated needle	Western medicine routine treatment	1 month	Not described	Not described	Not described	(1) Clinical effective rate. (2) Schober test outcome

Wang 2017 [31]	China	40	40	Superficial needling	Western medicine routine treatment	24 weeks	2	4	Not described	(1) TCM syndrome score

Dong 2017 [32]	China	30	30	Electrothermal needling	Western medicine routine treatment	8 weeks	Not described	Not described	Not described	(1) Clinical effective rate. (2) Schober test outcome

Chen 2017 [30]	China	54	54	Electroneedling	Western medicine routine treatment	3 months	Not described	Not described	Not described	(1) Clinical effective rate. (2) TCM syndrome score

Zhao 2016 [33]	China	60	60	Traditional needling treatment	Western medicine routine treatment	30 days	Not described	Not described	Not described	(1) Clinical effective rate

Zhang 2015 [34]	China	60	58	Traditional needling treatment	Western medicine routine treatment	30 days	Not described	Not described	Not described	(1) Clinical effective rate

Wang 2014 [35]	China	20	20	Superficial needling	Western medicine routine treatment	6 months	2	3	Not described	(1) Symptom score

Wei 2012 [36]	China	20	20	Fire needling	Western medicine routine treatment	4 weeks	Not described	Not described	Not described	(1) Clinical effective rate. (2) Symptom score

Zhang 2007 [37]	China	40	20	Traditional needling treatment	Western medicine routine treatment	4 months	Not described	Not described	Not described	(1) Clinical effective rate

Zhang 2005 [38]	China	23	20	Fire needling	Western medicine routine treatment	4 weeks	Not described	Not described	Not described	(1) Clinical effective rate. (2) Symptom score

## Abbreviations

AS: Ankylosing spondylitis
RCT: Randomized controlled trial
TCM: Traditional Chinese medicine
NSAIDs: Nonsteroidal anti-inflammatory drugs
TNFi: Tumor necrosis factor inhibitors
CI: Confidence interval
RR: Relative risk
MD: Mean difference
CNKI: China National Knowledge Infrastructure
CBM: Chinese Biomedical Literature Database
WF: Wangfang database
VIP: Chinese Science and Technology Periodical Database
MeSH: Medical Subject Heading.
